# Effects of bis-chelated copper in growth performance and gut health in broiler chickens subject to coccidiosis vaccination or coccidia challenge

**DOI:** 10.3389/fphys.2022.991318

**Published:** 2023-02-03

**Authors:** Juxing Chen, Frances Yan, Vivek A. Kuttappan, Karen Wedekind, Mercedes Vázquez-Añón, Deana Hancock

**Affiliations:** Novus International, Inc., St. Charles, MO, United States

**Keywords:** chelated copper, CuSO_4_, TBCC, gut microbiota, gut morphometry, broiler

## Abstract

Copper (Cu) is widely used at high levels as growth promoter in poultry, the alternative source of Cu to replace the high level of inorganic Cu at poultry farm remains to be determined. Three floor pen experiments were conducted to evaluate the effects of Cu methionine hydroxy-analogue chelate (Cu-MHAC, MINTREX^®^Cu, Novus International, Inc.) on growth performance and gut health in broilers in comparison to CuSO_4_ and/or tribasic copper chloride (TBCC). There were 3 treatments in experiment#1 (0, 30 and 75 ppm Cu-MHAC) and experiment#2 (15 and 30 ppm Cu-MHAC, and 125 ppm CuSO_4_), and 4 treatments in experiment #3 (15 and 30 ppm Cu-MHAC, 125 ppm CuSO_4_ and 125 ppm TBCC) with nine replicates pens of 10–13 birds in each treatment. The levels of other minerals were equal among all treatments within each experiment. All birds were orally gavaged with a coccidiosis vaccine at 1x recommended dose on d0 in experiment#1 and #2 and 10x recommended dose on d15 in experiment #3. Data were analyzed by one-way ANOVA, means were separated by Fisher’s protected LSD test. A *p* ≤ 0.05 was considered statistically different. In experiment #1, 30 and 75 ppm Cu-MHAC improved FCR during grower phase, increased jejunal villus height and reduced jejunal crypt depth, 30 ppm Cu-MHAC increased cecal *Lactobacillus spp.* abundance in 41 days broilers. In experiment #2, compared to CuSO_4_, 15ppm Cu-MHAC increased cumulative performance index in 28 days broilers, 15 and/or 30 ppm Cu-MHAC improved gut morphometry, and 30 ppm Cu-MHAC reduced the abundance of *E. coli* and *Enterobacteriaceae* in cecum in 43 days broilers. In experiment #3, 15 ppm and 30 ppm Cu-MHAC improved FCR *vs*. CuSO_4_ during starter phase, reduced the percentage of *E. coli* of total bacteria *vs*. TBCC, 30 ppm Cu-MHAC increased the percentages of *Lactobacillus acidophilus*, *Lactobacillus spp.* and *Clostridium cluster* XIVa of total bacteria *vs*. both CuSO_4_ and TBCC in the cecum of 27 days broilers. In summary, low doses of Cu-MHAC had comparable growth performance to high dose of TBCC and CuSO_4_ while improving gut microflora and gut morphometry in broilers subject to coccidiosis vaccination or coccidia challenge, indicating that low doses of bis-chelated Cu could be used as a complimentary strategy to improve animal gut health.

## Introduction

Copper (Cu) is widely used in concentrations in excess of the nutritional requirements as growth promoter and antimicrobial in poultry industry, however, the total amounts and concentrations of Cu used in feed may differ among countries due to different restrictions imposed by national legislation. The mechanism by which high levels of Cu promote growth remains to be determined. One of the possible mechanisms by which Cu may benefit birds is by shifting gut microbiota, thereby reducing susceptibility of birds to diseases and decreasing inflammation (Arias and Koutsos, 2006) and therefore increasing nutrient absorption (Hawbaker et al., 1961; Bunch et al., 1965). Although it’s commonly recognized that Cu exerts anti-microbial effect in the gut (Borkow and Gabbay, 2005; Borkow and Gabbay, 2009), there is not much direct and consistent data showing that Cu alters the population of bacterial species in poultry. Kim et al. (2011) showed that 100 ppm Cu-methionine and Cu-soy proteinate increased the population of *Lactobacillus* and decreased the population of *E. coli* and *Clostridium perfringens* in ileum, but 50 ppm Cu-methionine and Cu-soy proteinate were not effective. Pang et al. (2009) showed that 187.5 ppm CuSO_4_ and tribasic copper chloride (TBCC) increased *Lactobacilli* and decreased *E. Coli in vitro*, but did not impact the number of ileal *Lactobacilli* in birds. 150 ppm Cu_2_O decreased the abundance of some pathogenic bacterial families such as *Streptococcaceae* and *Corynebacteriaceae* and increased the abundance of some commensal bacteria like *Clostridiaceae* and *Peptostreptococcaceae* in broilers (Forouzandeh et al., 2021). More studies are needed to better understand how different sources of Cu modulate gut microbiota in broilers.

Cu is a cofactor of lysyl oxidase, an enzyme that crosslinks collagen subunits into mature protein forms to increase their strength, and it plays an important role in collagen crosslinking (Vallet and Ricard-Blum, 2019), therefore, Cu could help to maintain and improve the structural integrity of connective tissue such as skin, skeletal muscle, intestine and tendon (Richards et al., 2010). 100 and 200 ppm of Cu hydroxychloride supplementation increased jejunal villus height (Nguyen et al., 2022). Pharmacological levels (188 ppm) of CuSO_4_ and TBCC decreased the number of lamina propia lymphocytes and/or intraepithelial lymphocytes in duodenum and jejunum and duodenal crypt depth in comparison to negative control without Cu supplementation in birds raised on used litter, TBCC reduced jejunal crypt depth and increased ileal intraepithelial lymphocytes compared to antibiotics positive control and increased duodenal intraepithelial lymphocytes compared to negative control in broilers raised on fresh litter (Arias and Koutsos, 2006). These results suggest that Cu improved gut morphometry and reduced intestinal inflammation when birds were under “high” microbial environment challenge (Arias and Koutsos, 2006). Previous studies showed that Cu methionine hydroxy analog chelate (Cu-MHAC, MINTREX^®^Cu, Novus International, Inc.), a bis-chelated Cu, improved ileal breaking strength compared to Cu sulfate, Cu lysine and Cu proteinate (Richards et al., 2010), indicating that Cu could also improve mechanical property of intestine probably by promoting collagen cross-linking.

There is growing concern that heavy metals, such as Cu, could act as a selective pressure to force the proliferation and evolution of Cu-resistant and antibiotic-resistant bacteria at the farm and in the environment, both commensal and pathogenic enteral bacteria in farmed animals that developed resistance to trace elements such as Zn and Cu and concomitant cross-resistance to antimicrobial agents may be transferred to other animals and human (Yazdankhah et al., 2014). Trace elements such as Cu could also be toxic to bacteria due to their chemical affinity to the thiol groups of macro-biomolecules and their solubility under physiological conditions (Yazdankhah et al., 2014). Yu et al. (2017) commented that possible solutions to reduce the impact of heavy metal resistance include using organic trace mineral and reducing the supplemental levels of trace minerals in animal feed rations. In addition, the inclusion levels of Cu in complete poultry feed with a moisture level of 12% was approved to be less than 25 mg/kg feed in Europe Union (EU copper reduction plans officially approved, 2018) (https://www.allaboutfeed.net/animal-feed/feed-additives/eu-copper-reduction-plans-officially-approved), which indicates that highly bioavailable sources of Cu are needed to supplement at low levels to provide sufficient Cu for the growth of birds and in the meantime to comply the EU regulation.

Coccidiosis is a major intestinal challenge that causes economic loss to the broiler industry. The global cost of coccidiosis including losses during production and costs for prophylaxis and treatment in chickens is estimated to have been ∼ £10.36 billion at 2016 prices, which is equivalent to £0.16/chicken produced (Blake et al., 2020). In recent years, more and more poultry producers are looking into natural approaches to control coccidiosis, such as essential oils, probiotics, prebiotics, trace minerals, and other gut health enhancing products, which could be complimentary to the coccidiosis control programs. Chen et al. (2022) showed that supplementation of both bis-chelated mineral methionine hydroxy analog chelates (MMHAC) and high levels of total sulfur amino acid could overcome the growth performance challenges due to coccidiosis. It has been reported that Cu requirements were higher for chickens experiencing an acute phase response or pathological challenge compared to healthy chickens (Koh et al., 1996). For example, the chicks infected with *Eimeria (E.) tenella* had significantly elevated levels of serum copper and ceruloplasmin and liver copper than their pair-fed counterparts (Richards and Augustine., 1988). Different sources of organic and inorganic Cu sources are commercially available for poultry producers to use at different doses. Although there are numerous studies investigating the role of Cu supplementation in broilers subjected to coccidiosis (Rochell et al., 2017; Bortoluzzi et al., 2020; Dos Santos et al., 2020; Zaghari et al., 2022), the efficacy of different sources and doses of Cu supplementation in growth performance and gut health parameters of broilers subjected to coccidiosis vaccination or coccidia challenge is still inconsistent. The objective of this study was to determine an alternative source of Cu to replace high level of inorganic Cu in broilers subject to coccidiosis vaccination or coccidia challenge in poultry farm. Bis-chelated Cu, Cu-MHAC, has been reported to be a highly bioavailable Cu source (Wang et al., 2007), multiple low doses of Cu-MHAC were tested in three experiments to determine whether the low doses of Cu-MHAC could have similar growth performance as high dose of CuSO_4_ and/or TBCC while improving gut morphology and cecal microbiota in broilers subjected to coccidiosis vaccination or coccidia challenge.

## Materials and methods

### Birds and housing

Guide for the Care and Use of Agricultural Animals in Research and Teaching (FASS, 2010) was followed for housing and care of the animals throughout the experiments. All research procedures were reviewed and approved by the animal ethics committee composed of members from Novus International Inc (St. Charles, MO 63304) and a licensed veterinarian from Bridgeton Animal hospital (Bridgeton, MO 63044). Ross 308 male broilers were purchased from a local hatchery (Stover Hatchery, Stover, MO). Upon arrival, birds were placed immediately in 36 floor pens of 6 sqft with 10–13 birds per pen in an environmentally controlled room. Water was supplied to each pen *via* a nipple drinker system. Each pen had two drinkers and one hanging plastic feeder. Heat was furnished *via* natural gas fired radiant tube heaters and each pen was equipped with a brooding heat lamp. Test diets and water were provided for *ad libitum* consumption throughout the experiments. A 3 cm layer of used litter as bedding material was applied uniformly in each pen across all treatments on the first day of the experiment. The room was preheated to 27°C 2 days prior to study and kept at 27°C from 0 to 14 days. Room temperature was reduced to 26°C on d 14, 24°C on d 21, and 21°C on d 24 and kept at 21°C until the end of the experiment. For the first week, 23 h of light was provided. The light period was reduced to 20 h from d 8 to 39 and back to 23 h on d 40 until the end of the experiment.

### Experimental diets and performance data collection

Experimental diets were formulated to meet nutritional requirements of broiler chickens except for minerals. Composition and calculated nutrient profile of starter, grower, and finisher diets in three experiments were shown in Tables 1–3. Formulated and analyzed Cu concentration were shown in Table 4. For each phase, a common basal diet was made to reduce variation among test diets from weighing and mixing; aliquots of the basal diet were then supplemented with different sources of minerals. The methionine contribution from 2-hydroxy-4-methylthiobutyric acid (HMTBa) in Cu methionine hydroxy-analogue chelate (Cu-MHAC, MINTREX^®^Cu, Novus International, Inc.) was taken into consideration, and all final test diets were formulated to contain the same amount of supplemental HMTBa within each experiment. The starter diets were offered in crumbled, grower and finisher diets in pellet form with the pelleting temperature of 85°C. Birds were weighed by pen on d0, 14, 28 and 40 (Experiment #1) or d14, 28 and 42 (Experiment #2 and #3). On each weigh day other than d 0, feed consumption on pen basis was also determined. Mortality was checked and recorded twice daily, and weights of the dead birds were used to adjust feed conversion ratio (FCR). Cumulative Performance Index (cPI) was calculated as (cumulative livability*body weight*100/day of study)/cumulative FCR.

**TABLE 1:** Composition (%) and calculated nutrient content of basal diets during starter, grower and finisher phases in experiment #1.

Ingredients	Starter (d0-13)	Grower (d14-27)	Finisher (d28-41)
Soybean meal, 47.5% CP	28.07	29.73	24.06
Wheat, soft	44.64	37.79	43.37
Barley, 2 row	10.00	15.00	15.00
Rice bran	5.00	5.00	5.00
Poultry byproduct meal	2.03		
Choice white grease	5.23	8.35	8.27
L-lysine HCl	0.32	0.13	0.15
MHA—84%^a^	0.45	0.26	0.32
L-threonine	0.15	0.06	0.07
Dicalcium phosphate 18.5%	1.26	0.87	0.39
Limestone	1.31	1.44	1.60
Salt	0.25	0.26	0.26
Choline Chloride 60%	0.27	0.27	0.27
Sodium bicarbonate	0.46	0.29	0.29
Vitamin premix^b^	0.10	0.10	0.10
Santoquin mix6	0.02	0.02	0.02
Mold guard	0.05	0.05	0.05
Coban90	0.05	0.05	0.05
BMD-60	0.03	0.03	0.03
Mineral premix^c^	0.30	0.30	0.30
Total	100.0	100.0	100.0
Calc. nutrients			
ME, kcal/kg	3025	3150	3200
CP, %	22	21	19
Dig. Lys, %	1.27	1.10	0.97
Dig TSAA, %	0.94	0.84	0.76
Ca, %	1.05	0.90	0.85
Avail. P, %	0.45	0.33	0.23

^a^ DL-methionine hydroxy analogue calcium, MHA^®^ feed supplement (Novus International Inc., St Charles, MO).

^b^ Provided per kg of diet: vitamin A (from vitamin A acetate) 7001 IU; cholecalciferol 2750 IU; vitamin E (from vitamin E acetate) 33 IU; vitamin B_12_ 0.014 mg; riboflavin 6.5 mg; niacin 37.5 mg; pantothenate 10 mg (from calcium pantothenate); Vitamin K (from menadione sodium bisulfite) 2.01 mg; folic acid 0.9 mg; thiamin (from thiamin mononitrate) 1.8 mg; pyridoxine (from pyridoxine HCl) 3.5 mg; d-biotin 0.15 mg.

^c^ Provided per kg of diet: Mn (from MnSO_4_·H_2_O) 60 mg; Zn (from ZnSO_4_·H_2_O) 40 mg; Fe (from FeSO_4_·H_2_O) 80 mg; Cu (from CuSO_4_·5H_2_O) 8 mg; I from CaI_2_O_6_, 1.25 mg; and Se 0.15 mg (from Na_2_SeO_3_).

**TABLE 2:** Composition (%) and calculated nutrient content of basal diets during starter, grower and finisher phases in experiment #2.

Ingredients	Starter (d0-13)	Grower (d14-27)	Finisher (d28-43)
Soybean meal, 47.5%CP	29.07	29.48	23.4
Wheat, soft	44.76	38.72	45.07
Barley, 2 row	10.0	15.0	15.0
Rice bran	5.0	5.0	5.0
Poultry byproduct meal	3.04	0	0
Choice white grease	4.57	7.92	7.85
L-lysine HCl	0.25	0.13	0.15
MHA—84%^a^	0.41	0.34	0.31
L-threonine	0.15	0.09	0.10
Dicalcium phosphate 18.5%	0.28	0.50	0.23
Limestone	1.33	1.23	1.30
Salt	0.21	0.23	0.23
Choline Chloride 60%	0.26	0.27	0.26
Sodium bicarbonate	0.32	0.34	0.35
Vitamin premix^b^	0.050	0.050	0.050
Phytase^c^	0.005	0.005	0.005
TiO2	0	0.40	0.40
Mineral premix^d^	0.30	0.30	0.30
Total	100.0	100.0	100.0
Calculated nutrients			
ME, kcal/kg	3025	3150	3200
CP, %	22.0	21.0	19.0
Dig. Lys, %	1.27	1.10	0.97
Dig TSAA, %	0.94	0.84	0.76
Ca, %	1.05	0.90	0.85
Avail. P, %	0.45	0.33	0.23

^a^ DL-methionine hydroxy analogue calcium, MHA^®^ feed supplement (Novus International Inc., St Charles, MO).

^b^ Provided per kg of diet: vitamin A (from vitamin A acetate) 7001 IU; cholecalciferol 2750 IU; vitamin E (from vitamin E acetate) 33 IU; vitamin B_12_ 0.014 mg; riboflavin 6.5 mg; niacin 37.5 mg; pantothenate 10 mg (from calcium pantothenate); Vitamin K (from menadione sodium bisulfite) 2.01 mg; folic acid 0.9 mg; thiamin (from thiamin mononitrate) 1.8 mg; pyridoxine (from pyridoxine HCl) 3.5 mg; d-biotin 0.15 mg.

^c^ CIBENZA^®^ PHATEVERSE^®^ G10 (Novus International Inc., St Charles, MO): provided minimum 10,000 units of phytase per gram of product.

^d^ Provided per kg of diet: Mn (from MnSO_4_·H_2_O) 60 mg; Zn (from ZnSO_4_·H_2_O) 40 mg; Fe (from FeSO_4_·H_2_O) 80 mg; Cu (from CuSO_4_·5H_2_O) 8 mg; I from CaI_2_O_6_, 1.25 mg; and Se 0.15 mg (from Na_2_SeO_3_).

**TABLE 3:** Composition (%) and calculated nutrient content of basal diets during starter, grower and finisher phases in experiment #3.

Ingredients	Starter (d0-13)	Grower (d14-27)	Finisher (d28-42)
Corn	60.67	62.99	67.26
SBM	28.25	22.84	16.57
DDGS	5.0	7.5	10.0
Meat and bone meal	3.0	3.0	1.88
Choice white grease	0.34	1.31	1.94
Limestone	0.88	0.82	0.93
Salt	0.42	0.41	0.43
Dicalcium phosphate	0.36	0.03	0
L-Lysine HCl 78%	0.27	0.27	0.25
MHA—84%^a^	0.27	0.21	0.13
Trace mineral premix^b^	0.20	0.20	0.20
Threonine	0.10	0.08	0.06
Choline Cl-60%	0.07	0.07	0.08
Vitamin premix^c^	0.050	0.050	0.050
Phytase^d^	0.005	0.005	0.005
Oro Glo 20 (pigment)	0	0.10	0.10
Additives/corn	0.12	0.12	0.12
Total	100.0	100.0	100.0
Calculated nutrient profile			
ME, kcal/kg	3025	3120	3200
Crude protein, %	22.23	20.49	17.89
Lys, digestible %	1.21	1.08	0.90
Met, digestible %	0.59	0.52	0.42
TSAA, digestible %	0.91	0.82	0.69

^a^ DL-methionine hydroxy analogue calcium, MHA^®^ feed supplement (Novus International Inc., St Charles, MO).

^b^ Provided per kg of diet: Mn (from MnSO_4_·H_2_O) 60 mg; Zn (from ZnSO_4_·H_2_O) 40 mg; Fe (from FeSO_4_·H_2_O) 80 mg; Cu (from CuSO_4_·5H_2_O) 8 mg; I from CaI_2_O_6_, 1.25 mg; and Se 0.15 mg (from Na_2_SeO_3_).

^c^ Provided per kg of diet: vitamin A (from vitamin A acetate) 7001 IU; cholecalciferol 2750 IU; vitamin E (from vitamin E acetate) 33 IU; vitamin B_12_ 0.014 mg; riboflavin 6.5 mg; niacin 37.5 mg; pantothenate 10 mg (from calcium pantothenate); Vitamin K (from menadione sodium bisulfite) 2.01 mg; folic acid 0.9 mg; thiamin (from thiamin mononitrate) 1.8 mg; pyridoxine (from pyridoxine HCl) 3.5 mg; d-biotin 0.15 mg.

^d^ CIBENZA^®^ PHATEVERSE^®^ G10 (Novus International Inc., St Charles, MO): provided minimum 10,000 units of phytase per gram of product.

^e^ ORO GLO^®^ (Kemin Industries Inc., Des moines, IA): provided 20 g of xanthophylls per kg of product.

**TABLE 4:** Formulated and analyzed Cu concentrations in three experiments.

Formulated Cu concentration (ppm)		Analyzed Cu concentrations (ppm)		
		Starter	Grower	Finisher
Experiment #1				
Control	0	11.4	9.6	8.2
Cu-MHAC-30 ppm	30	48.1	34.5	37.0
Cu-MHAC-75 ppm	75	88.1	83.0	90.8
Experiment #2				
Cu-MHAC, 15 ppm	15	21.8	16.8	20.6
Cu-MHAC, 30 ppm	30	42.9	38.7	29.0
CuSO_4_, 125 ppm	120	112.0	105.0	114.0
Experiment #3				
CuSO_4_, 125 ppm	125	121.0	111.0	107.0
Cu-MHAC, 15 ppm	15	44.1	18.3	27.3
Cu-MHAC, 30 ppm	30	42.1	29.5	37.1
TBCC, 125 ppm	125	133.0	113.0	111.0

### Experimental design

#### Experiment #1

A total of 351 day-old Ross 308 male broilers were randomly assigned to one of the 3 treatments: 0, 30 or 75 ppm Cu-MHAC (MINTREX^®^Cu, Novus International, Inc.). Supplemental levels of Zn (32 ppm) and Mn (32 ppm) from MHAC (MINTREX^®^Zn:Mn, Novus International, Inc.), Fe (40 ppm), I (1.25 ppm), and Se (0.3 ppm) from inorganic sources were equal among 3 treatments. Each diet was fed to nine replicate pens of 13 broilers with randomized complete block design. Broilers were vaccinated with 1× recommended dose of coccidiosis vaccine (mixed species of *E. acervulina*, *E. tenella*, and *E. maxima* from Huvepharma, Inc.) by oral gavage on d0. On d41, 1 bird/pen was sacrificed to collect duodenum and jejunum tissue for gut morphometry measurement and cecal content for bacteria quantitation.

#### Experiment #2

A total of 270 day-old Ross 308 male broilers were randomly assigned to one of the 3 treatments: 15 or 30 ppm Cu-MHAC (MINTREX^®^Cu, Novus International, Inc) or 125 ppm CuSO_4_. Supplemental levels of Zn (100 ppm), Mn (90 ppm), Fe (40 ppm), I (1.25 ppm), and Se (0.3 ppm) from inorganic sources were equal among 3 treatments. Each diet was fed to nine replicate pens of 10 broilers with randomized complete block design. Broilers were vaccinated with 1× recommended dose of coccidiosis vaccine as described above in Experiment #1 by oral gavage on d0. On d43, 1 bird/pen was sacrificed to collect duodenum, jejunum and ileum tissue for gut morphometry measurement and cecal content for bacteria quantitation.

#### Experiment #3

A total of 468 day-old Ross 308 male broilers were randomly assigned to one of the 4 treatments: 15 or 30 ppm Cu-MHAC (MINTREX^®^Cu, Novus International, Inc.), 125 ppm CuSO_4_, or 125 ppm TBCC. Supplemental levels of Zn (100 ppm), Mn (90 ppm), Fe (40 ppm), I (1.25 ppm), and Se (0.3 ppm) from inorganic sources were equal among 3 treatments. Each diet was fed to nine replicate pens of 13 broilers with randomized complete block design. On d15, broilers were challenged with 10× recommended dose of coccidiosis vaccine as described above in Experiment #1 by oral gavage. On d29, 1 bird/pen was sacrificed to collect cecal content for bacteria quantitation.

#### Histological sample preparation and gut morphometry measurements

Duodenum, jejunum and ileum tissue were taken, flushed with formalin-free histology fixative (NOTOXhisto Fixative; Scientific Device Laboratory; Des Plaines. IL) and fixed with NOTOXhisto for 4 weeks. Fixed intestine tissue was trimmed in cross-sections, processed, embedded in paraffin and sliced in cross-sections. A 5 μm section of each sample was placed on a glass slide and stained with hematoxylin and eosin for morphometry examination and measurement under Olympus light microscope. In each intestine cross-section, five replicates of each variable were measured from each sample using ZEN lite 2012 Imaging software (ZEISS Microscopy; Thornwood, NY). A total of representative 5 villus and 5 crypts in each slide were selected to measure villus height, villus width, crypt depth. Muscularis layer thickness, which includes muscularis propria, subserosa and serosa, was measured at 5 representative locations of each intestine slide. Villus height was measured from the top of the villus to the top of the lamina propria. Crypt depth was measured from the base upwards to the region of transition between the crypt and villus. Villus width was measured at the middle of each villus, whereas crypt depth to villus height ratio (CVR) was determined as the ratio of crypt depth to villus height, while villus height to villus width ratio (HWR) was determined as the ratio of villus height to villus width.

#### Cecal bacteria quantitation

Cecal content was collected from each bird and snap-frozen in liquid nitrogen and stored at -20°C freezer until sample analysis. Genomic DNA was extracted from cecal content using PowerLyzer^®^ PowerSoil^®^ DNA Isolation Kit (Qiagen, Germantown, MD). DNA concentration was quantified and 8 ng of cecal DNA was used to measure the abundance of bacteria by quantitative polymerase chain reaction (qPCR) using SYBR Green in the QuantStudio 5 RealTime PCR System (Applied Biosystems; Foster City, CA) in a 384-well plate and primers described in Table 5. All primers were verified for efficiency (90% ± 10%) and linearity of amplification (r^2^ ≥ 0.99). The abundance of different bacteria per ng DNA from cecal contents at family or specie levels was expressed as Ct value per ng DNA, lower Ct means higher abundance, and *vice versa*, higher Ct means lower abundance. The relative abundance of bacteria was expressed as the percentage % of total bacteria by calculating delta Ct of different bacteria *verse (vs)* total bacteria with 1 Ct difference equaling 2-fold difference in bacterial abundance.

**TABLE 5:** Sequence of primers used in this study.

Bacteria	Forward sequence (5′-3′)	Reverse sequence (5′-3′)
Total bacteria (domain)	ACT​CCT​ACG​GGA​GGC​AGC​AG	ATTACCGCGGCTGCTGG
Enterobacteriaceae (family)	CAT​TGA​CGT​TAC​CCG​CAG​AAG​AAG​C	CTC​TAC​GAG​ACT​CAA​GCT​TGC
*E. coli* subgroup (species)	GTT​AAT​ACC​TTT​GCT​CAT​TGA	ACC​AGG​GTA​TCT​AAT​CCT​GT
*L. acidophilus* (species)	AGA​GGT​AGT​AAC​TGG​CCT​TTA	GCGGAAACCTCCCAACA
*Lactobacillus* spp (species)	AGC​AGT​AGG​GAA​TCT​TCC​A	CACCGCTACACATGGAG
*C. clusterXIVa* (species)	GAWGAAGTATYTCGGTATCT	CTACGCWCCCTTTACAC

#### Data analysis

All data were subjected to analysis of variance as a randomized complete block design using the PROC MIXED procedure of SAS 9.4. Pen was used as the experimental unit for the analysis. Data were analyzed by one-way ANOVA, means were separated by Fisher’s protected LSD test. The incidences of wooden breast were analyzed by Chi-square test. A *p*-value ≤ 0.05 was considered statistically different. A *p*-value 0.05< *p* ≤ 0.10 was considered a numerical trend.

## Results

### Experiment #1

In experiment #1, 30 ppm and 75 ppm Cu-MHAC supplementation improved period FCR by 2.8 and 2.6 points, respectively, during grower phase (d15-27) in comparison to control without Cu supplementation and had no effect on other growth performance parameters at different time points (Table 6). Compared to control, 30 ppm and 75 ppm Cu-MHAC increased (*p* < 0.05) villus height and HWR and reduced (*p* < 0.05) crypt depth and CVR in jejunum but not in duodenum (Table 7).

**TABLE 6:** Growth performance of broilers in experiment #1.

Treatment	Body weight (kg)	Period FCR (kg/kg)	cFCR (kg/kg)	cFI (kg)	cPI
d0					
Control	0.041				
Cu-MHAC-30 ppm	0.041				
Cu-MHAC-75 ppm	0.041				
SEM	0.0003				
*p*-value	0.8582				
d14					
Control	0.536	1.203	1.203	0.595	318.5
Cu-MHAC-30 ppm	0.527	1.202	1.202	0.585	313.3
Cu-MHAC-75 ppm	0.530	1.220	1.220	0.597	310.4
SEM	0.007	0.010	0.010	0.006	6.5
*p*-value	0.4563	0.1440	0.1440	0.2160	0.3711
d28					
Control	1.603	1.476^a^	1.378	2.152	430.4
Cu-MHAC-30 ppm	1.582	1.448^b^	1.360	2.093	428.0
Cu-MHAC-75 ppm	1.598	1.450^b^	1.369	2.131	432.4
SEM	0.048	0.009	0.008	0.052	15.6
*p*-value	0.7411	0.0484	0.2374	0.2591	0.9105
d40					
Control	3.189	1.699	1.514	4.768	519.1
Cu-MHAC-30 ppm	3.207	1.713	1.503	4.755	522.0
Cu-MHAC-75 ppm	3.160	1.765	1.511	4.709	515.5
SEM	0.101	0.044	0.009	0.136	17.5
*p*-value	0.7946	0.3035	0.5867	0.8223	0.9121

cFCR, Cumulative FCR; cFI, Cumulative feed intake; cPI: Cumulative performance index.

Different superscript letters. ^a,b^ show significant differences (*p* ≤ 0.05) between treatments means ± SEM within each time point for parameters measured.

**TABLE 7:** Duodenal and jejunal morphometry of broilers at 41 days of age.

Treatment	Villus height (μm)	Villus width (μm)	Crypt depth (μm)	Muscularis thickness (μm)	HWR^a^	CVR^b^
Duodenum						
Control	2655.772	184.777	90.207^ab^	284.351	15.430	0.034^xy^
Cu-MHAC-30 ppm	2543.393	206.587	79.907^b^	258.772	12.586	0.032^y^
Cu-MHAC-75 ppm	2653.639	190.899	100.010^a^	270.178	14.668	0.038^x^
SEM	87.979	15.102	4.700	20.725	1.199	0.002
*p*-value	0.5932	0.5813	0.0207	0.6863	0.2417	0.0639
Jejunum						
Control	1165.189^b^	141.538	104.863^a^	287.022	8.542^b^	0.091^a^
Cu-MHAC-30 ppm	1837.322^a^	160.227	91.084^b^	234.434	12.937^a^	0.050^b^
Cu-MHAC-75 ppm	1852.820^a^	129.03	90.090^b^	268.677	14.684^a^	0.049^b^
SEM	65.184	12.699	3.824	16.944	1.208	0.003
*p*-value	<0.0001	0.2372	0.0194	0.1048	0.0043	<0.0001

^a^ CVR: the ratio of crypt depth to villus height.

^b^ HWR: the ratio of villus height to villus width.

Different superscript letters. ^a,b^ show significant differences (*p* ≤ 0.05) between treatments means ± SEM within each tissue for parameters measured.

Different superscript letters. ^x,y^ show numerical trend (0.05 < *p* ≤ 0.10) between treatments means ± SEM within each tissue for parameters measured.

Cecal DNA was extracted to measure bacterial population by qPCR. Supplementation of 30 ppm Cu-MHAC significantly (*p* < 0.05) decreased the Ct of *Lactobacillus spp* per ng of cecal DNA (Table 8), indicating that it increased the abundance of *Lactobacillus spp* in cecum. No differences in the abundance and relative abundance of other bacteria were detected between treatments. It’s unexpected that 75 ppm Cu-MHAC did not alter the abundance of *Lactobacillus spp* and *Lactobacillus acidophilus*.

**TABLE 8:** Ct of *E. coli*, *Lactobacillus acidophilus*, *Lactobacillus spp*. and *Enterobacteriaceae* per ng of cecal DNA, and percentage (%) of *E. Coli*, *Lactobacillus L.) Acidophilus*, *L.spp*. and *Enterobacteriaceae* (% of total bacteria) in cecum of broilers at 41 days of age of experiment #1.

Treatment	% Of bacterial abundance in total bacteria				Ct of bacteria per ng of cecal DNA				
	*E. coli (Species)*	*Enterobact-eriaceae (Family)*	*L. Acidophilus (Species)*	*L. spp (Species)*	*Ecoli (Species)*	*Enterobact-eriaceae (Family)*	*L. Acidophilus (Species)*	*L. spp (Species)*	Total bacteria
Control	1.3	1.3	1.6	7.3	23.789	22.810	22.219	20.267^a^	15.535
Cu-MHAC, 30 ppm	1.8	1.7	4.8	11.3	22.397	21.565	20.844	18.234^b^	14.455
Cu-MHAC, 75 ppm	4.3	3.7	2.2	7.3	23.930	22.699	21.646	20.730^a^	15.478
SEM	1.6	1.2	1.5	3.0	1.309	1.047	1.172	0.784	0.525
*p*-value	0.3914	0.3619	0.3165	0.3162	0.6613	0.6491	0.6868	0.0158	0.0788

Different superscript letters. ^a,b^ show significant differences (*p* ≤ 0.05) between treatments means ± SEM for parameters measured.

### Experiment #2

There was no significant treatment difference of growth performance during all phases except for the improvement (*p* < 0.04) of d28 cumulative performance index by supplementation of 15 ppm Cu-MHAC in comparison to 125 ppm CuSO_4_, which resulted from the numerical increase of body weight and cumulative FCR (Table 9).

**TABLE 9:** Growth performance of broilers in experiment #2.

Treatment	Body weight (kg)	Period FCR (kg/kg)	cFCR (kg/kg)	cFI (kg)	cPI
d0					
Cu-MHAC, 15 ppm	0.043				
Cu-MHAC, 30 ppm	0.043				
CuSO_4_, 125 ppm	0.043				
SEM	0.0003				
*p*-value	0.7427				
d14					
Cu-MHAC, 15 ppm	0.515	1.231	1.231	0.581	279.3
Cu-MHAC, 30 ppm	0.518	1.230	1.230	0.585	277.8
CuSO_4_, 125 ppm	0.506	1.235	1.235	0.571	270.0
SEM	0.006	0.011	0.011	0.007	4.2
*p*-value	0.2138	0.9326	0.9326	0.3571	0.2075
d28					
Cu-MHAC, 15 ppm	1.630	1.502	1.406	2.232	414.4^a^
Cu-MHAC, 30 ppm	1.601	1.546	1.433	2.232	390.0^b^
CuSO_4_, 125 ppm	1.588	1.523	1.422	2.196	394.5^b^
SEM	0.019	0.017	0.012	0.024	5.1
*p*-value	0.3049	0.1494	0.1686	0.4824	0.0047
d42					
Cu-MHAC, 15 ppm	3.218	1.874	1.626	5.165	458.3
Cu-MHAC, 30 ppm	3.163	1.926	1.665	5.182	437.9
CuSO_4_, 125 ppm	3.214	1.877	1.647	5.212	459.8
SEM	0.076	0.048	0.019	0.092	13.9
*p*-value	0.8454	0.6894	0.3763	0.9396	0.4388

cFCR: cumulative FCR; cFI: cumulative feed intake; cPI: cumulative performance index.

Different superscript letters.

^a,b^ show significant differences (*p* ≤ 0.05) between treatments means ± SEM within each time point for parameters measured.

In birds at 42 days of age, compared to 125 ppm CuSO_4_, 15 ppm Cu-MHAC reduced the thickness of muscularis layer in jejunum (*p* = 0.0243) and ileum (*p* = 0.0424), and crypt depth in ileum (*p* = 0.0493); 30 ppm Cu-MHAC reduced the muscular layer thickness in duodenum (*p* = 0.0405) (Table 10).

**TABLE 10:** Duodenal, jejunal and ileal morphometry of broilers at 43 days of age.

Treatment	Villus height (μm)	Villus width (μm)	Crypt depth (μm)	Muscularis thickness (μm)	HWR^a^	CVR^b^
Duodenum						
Cu-MHAC, 15 ppm	2379.739	180.412	85.467	241.713^ab^	13.687	0.036
Cu-MHAC, 30 ppm	2416.827	175.479	86.344	212.638^b^	14.232	0.036
CuSO_4_, 125 ppm	2384.675	208.646	90.010	286.564^a^	12.188	0.038
SEM	108.610	13.959	4.066	19.411	1.345	0.002
*p*-value	0.9649	0.2188	0.7146	0.0405	0.5474	0.7836
Jejunum						
Cu-MHAC, 15 ppm	1446.833	150.084	90.700	219.866^b^	9.663	0.063
Cu-MHAC, 30 ppm	1570.028	145.561	89.338	261.120^ab^	11.052	0.057
CuSO_4_, 125 ppm	1653.327	162.313	90.160	305.631^a^	10.443	0.055
SEM	71.580	7.988	5.782	20.018	0.697	0.004
*p*-value	0.1553	0.3265	0.9856	0.0243	0.3794	0.3021
Ileum						
Cu-MHAC, 15 ppm	1070.550	135.016	76.487^b^	275.829^b^	8.074	0.072
Cu-MHAC, 30 ppm	1165.957	134.507	87.976^a^	313.266^ab^	8.742	0.077
CuSO_4_, 125 ppm	1173.491	144.103	88.429^a^	354.351^a^	8.318	0.076
SEM	54.977	8.372	3.600	20.135	0.452	0.004
*p*-value	0.3619	0.6689	0.0493	0.0424	0.5701	0.7382

^a^ CVR: The ratio of crypt depth to villus height.

^b^ HWR: The ratio of villus height to villus width.

Different superscript letters. ^a,b^ show significant differences (*p* ≤ 0.05) between treatments means ± SEM within each tissue for parameters measured.

Compared with 125 ppm CuSO_4_, 30 ppm Cu-MHAC significantly (*p* < 0.05) reduced the abundance of *Escherichia coli* (*E. coli)* and *Enterobacteriaceae* in cecum of birds of 42 days of age (Table 11).

**TABLE 11:** Ct of *Firmicutes*, *E. Coli*, *Lactobacillus Acidophilus*, *Lactobacillus spp*. and *Enterobacteriaceae* per ng of cecal DNA, and percentage (%) of *Firmicutes*, *E. Coli*, *Lactobacillus Acidophilus*, *Lactobacillus spp*. and *Enterobacteriaceae* (% of total bacteria) in broilers at 43 days of age of experiment #2.

Treatment	% Of bacterial abundance in total bacteria				Ct of bacteria per ng of cecal DNA				
	*Ecoli (Species)*	*Enterobact-eriaceae (Family)*	*L. Acidophilus (Species)*	*L. spp (Species)*	*Ecoli (Species)*	*Enterobact-eriaceae (Family)*	*L. Acidophilus (Species)*	*L. spp (Species)*	*Total bacteria*
Cu-MHAC, 15 ppm	0.057	0.083	0.146	2.952	27.331^ab^	26.686^ab^	25.668	20.443	15.134
Cu-MHAC, 30 ppm	0.016	0.022	0.130	1.981	29.150^a^	28.950^a^	25.271	23.498	15.602
CuSO4, 125 ppm	0.048	0.109	0.144	2.207	26.237^b^	24.949^b^	25.300	20.691	14.978
SEM	0.017	0.033	0.052	0.544	0.747	0.838	1.078	1.633	0.376
*p*-value	0.161	0.0884	0.9469	0.2237	0.0348	0.0114	0.944	0.3708	0.4895

Different superscript letters. ^a,b^ show significant differences (*p* ≤ 0.05) between treatments means ± SEM for parameters measured.

### Experiment #3

15 and 30 ppm of Cu-MHAC significantly improved (*p* < 0.05) d0-14 FCR by 2.8 and 3 points, respectively, compared to CuSO_4_ treatment, TBCC was intermediate (Table 12). No significant difference was observed for body weight, period FCR, cumulative FCR, feed intake and performance index on d14. Neither dose of Cu-MHAC improved growth performance parameters during grower and finisher phase (Table 12).

**TABLE 12:** Growth performance of broilers in experiment #3.

Treatment	Body weight (kg)	Period FCR (kg/kg)	cFCR (kg/kg)	cFI (kg)	cPI
d0					
CuSO_4_, 125 ppm	0.036				
Cu-MHAC, 15 ppm	0.036				
Cu-MHAC, 30 ppm	0.036				
TBCC, 125 ppm	0.036				
SEM	0.0003				
*p*-value	0.5599				
d14					
CuSO_4_, 125 ppm	0.499	1.237^a^	1.237^a^	0.572	287.7
Cu-MHAC, 15 ppm	0.516	1.209^b^	1.209^b^	0.580	301.8
Cu-MHAC, 30 ppm	0.508	1.207^b^	1.207^b^	0.569	298.7
TBCC, 125 ppm	0.506	1.225^ab^	1.225^ab^	0.575	295.2
SEM	0.005	0.007	0.007	0.005	4.0
*p*-value	0.1513	0.017	0.017	0.4988	0.103
d28					
CuSO_4_, 125 ppm	1.458	1.606	1.485	2.111	360.0
Cu-MHAC, 15 ppm	1.490	1.620	1.484	2.157	369.0
Cu-MHAC, 30 ppm	1.457	1.633	1.491	2.118	359.3
TBCC, 125 ppm	1.458	1.648	1.507	2.143	352.1
SEM	0.014	0.014	0.009	0.021	5.2
*p*-value	0.2543	0.1070	0.1500	0.4107	0.1390
d42					
CuSO_4_, 125 ppm	3.074	1.947	1.676	5.088	429.6
Cu-MHAC, 15 ppm	3.170	1.914	1.664	5.212	450.1
Cu-MHAC, 30 ppm	3.108	1.917	1.67	5.131	432.0
TBCC, 125 ppm	3.155	1.902	1.675	5.224	437.0
SEM	0.042	0.025	0.010	0.069	8.6
*p*-value	0.3676	0.577	0.798	0.4558	0.356

cFCR: cumulative FCR; cFI: cumulative feed intake; cPI: cumulative performance index. Different superscript letters. ^a,b^ show significant differences (*p* ≤ 0.05) between treatments means ± SEM within each time point for parameters measured.

The abundance of different bacteria including total bacteria per ng DNA of cecal contents was not different among 4 treatments (Table 13). The relative abundance of these bacteria expressed as their percentage in total bacteria were also compared among 4 treatments (Table 13). 30 ppm Cu-MHAC increased (*p* < 0.05) the relative abundance of *Lactobacillus acidophilus*, *Lactobacillus spp*. and *Clostridium cluster XIVa* in comparison to CuSO_4_ and TBCC treatments. Both 15 and 30 ppm Cu-MHAC decreased (*p* < 0.05) the relative abundance of *E. Coli* compared to TBCC, but not different from CuSO_4_ treatment. The relative abundance of *Firmicutes* and *Bacteroidetes* at phylum levels were not different among 4 treatments.

**TABLE 13:** The Ct of *E. coli*, *Lactobacillus acidophilus*, *Lactobacillus spp*. and *Clostridium cluster XIVa* (% of total bacteria) per ng of cecal DNA and the relative population (%) of *E. coli*, *Lactobacillus acidophilus*, *Lactobacillus spp*. and *Clostridium cluster XIVa* (% of total bacteria) in the cecum of broilers at 29 days of age in experiment #3.

Treatment	*E. coli* (Species)	*Enterobacteriaceae* (Family)	*L. acidophilus* (Species)	*L. spp* (Species)	*Clostridium cluster XIVa* (Species)	Total bacteria
	Ct	Ct	Ct	Ct	Ct	Ct
CuSO_4_, 125 ppm	27.820	24.341	22.922	21.393	19.670	13.583
Cu-MHAC, 15 ppm	26.150	21.914	22.294	20.748	19.912	13.579
Cu-MHAC, 30 ppm	25.720	21.915	21.907	20.474	19.328	13.770
TBCC, 125 ppm	26.440	23.070	23.397	21.920	20.150	13.812
SEM	1.162	0.952	0.763	0.638	0.275	0.107
*p*-value	0.6120	0.2460	0.5334	0.3932	0.1475	0.2922
Treatment	*%*	*%*	*%*	*%*	*%*	
CuSO_4_, 125 ppm	0.020^b^	0.462	0.289^b^	0.643^b^	1.109^b^	
Cu-MHAC, 15 ppm	0.099^b^	2.583	0.111^b^	0.417^b^	1.056^b^	
Cu-MHAC, 30 ppm	0.119^b^	4.333	0.722^a^	1.406^a^	1.914^a^	
TBCC, 125 ppm	0.537^a^	2.819	0.120^b^	0.304^b^	0.964^b^	
SEM	0.130	1.141	0.167	0.295	0.241	
*p*-value	0.0320	0.1612	0.0360	0.0510	0.0310	

Different superscript letters. ^a,b^ show significant differences (*p* ≤ 0.05) between treatments means ± SEM for parameters measured.

## Discussion

### Low dose of Cu-MHAC supplementation had comparable growth performance as high dose of CuSO_4_ and TBCC

In all three experiments, Cu-MHAC improved a few growth performance parameters during either starter or grower phases, but not during finisher phase in *Eimeria* vaccinated or challenged birds.

Cu is required for the development and maintenance of immune system and Cu deficiency influences the ability of animals to maintain their immunity (Percival, 1998). Oxidative stress and inflammation are frequently involved in enteric diseases of broilers, and they are part of normal defense mechanisms against pathogens (Lauridsen, 2019). Inflammation is a generic response that is considered as a mechanism of innate immunity fighting against pathogens (Lauridsen, 2019). Individuals suffering from oxidative stress might correspondingly have weak immune responses in order to minimize oxidative damage (Cram et al., 2015).

Cu-MHAC is more bioavailable than ITM (Wang et al., 2007) and MMHAC has been reported to improve antioxidant status in broilers and lactating Holstein cow and vaccine-induced anti-M. hyopneumoniae antibody titers in gilts (Richards et., al., 2010; Zhao et al., 2015). On the other hand, inorganic Cu especially when supplemented at high levels could become pro-oxidants and increase reactive oxygen species and malondialdehyde leading to oxidative stress (Yang et al., 2019). Song et al., (2021) reported that immune system and its function are not well developed from d6 to d13, and not mature until d30 to 34 in the broiler chickens in cages, and it is necessary to enhance the immune function of the broiler chickens through nutritional supplementation from d1 to 30. In our studies, birds were reared in floor pens, and they would develop immunity to *Eimeria* after a few cycles of *Eimeira* infection, it’s possible that Cu-MHAC either improved immunity development before *Eimeria* challenge during starter phase or boosted immunity during grower phase, therefore, the growth performance was improved to a certain degree during starter and/or grower phase but not during finisher phase when birds gained full immunity against *Eimeira*. These hypotheses warrant further investigation in future studies.

Overall, low dose of Cu-MHAC supplementation had similar growth performance as high dose of CuSO_4_ and TBCC in broilers.

### Cu-MHAC improved gut morphometry

The villi-crypt unit in intestinal epithelium is responsible for nutrient absorption (GÜunther et al., 2013). The epithelial cells near the villous tip have the strongest digestion and absorption ability of nutrients (Hampson, 1986), therefore, more epithelial cells and longer villus height could increase nutrients absorption (Thomson and Keelean, 1986), increase of villus height and villus height/villus width ratio indicates greater villus absorption capacity, and *vice versa*, decrease of villus height and villus height/villus width ratio indicates lower villus absorption capacity. For example, the significant decrease of villi length contributes to reduced digestive capacity in postweaning piglets (Montagne et al., 2007). In study #1, 30 ppm and 75 ppm Cu-MHAC increased (*p* < 0.05) jejunal villus height and villus height/villus width ratio, suggesting that Cu-MHAC probably improved jejunal villus absorption capacity. Consistent with this study, Nguyen et al. (2022) reported that feeding 100 and 200 ppm Cu hydroxychloride increased jejunal villus height in broilers.

The enterocyte in intestinal epithelium has high turnover rate and renews itself every 4–5 days, the enterocyte turnover occurs with cell loss in the intestinal lumen due to apoptosis of epithelial cells near the villous tip, then the cells in the crypts will proliferate and migrate towards the apex of the villi to replace the cell loss (GÜunther et al., 2013). Therefore, decreased crypt depth and crypt depth/villus height ratio indicate slower enterocyte turnover, which would reduce the nutrient and energy needed for gut maintenance and reserve more nutrient and energy for animal growth and tissue development. 30 and 75 ppm Cu-MHAC reduced (*p* < 0.05) jejunal crypt depth and crypt depth/villus height ratio in experiment #1. 15 ppm Cu-MHAC reduced ileal crypt depth in experiment #2. These results suggest that Cu-MHAC likely slowed down the enterocyte turnover which could help save nutrients and energy for potential animal growth and tissue development.

Thickening of muscularis propria has been reported to be positively correlated with chronic inflammation in Crohn’s disease patients (Chen et al., 2017). 15 and/or 30 ppm Cu-MHAC decreased the thickness of muscularis layer in multiple sections of intestine compared to CuSO_4_ in experiment #2 suggesting that Cu-MHAC probably reduced intestinal inflammation.

Collectively, dietary inclusion of Cu-MHAC in broiler diets improved gut structural integrity with greater villus height, shorter crypt depth and/or thinner muscularis layer thickness.

### Cu-MHAC modulated gut microbiota

One of the possible mechanisms by which Cu may benefit birds is by shifting the gut microbiota, thereby reducing susceptibility of birds to diseases and decreasing intestinal lymphocyte recruitment and infiltration (Arias and Koutsos, 2006) and thus increasing nutrient absorption (Hawbaker et al., 1961; Bunch et al., 1965). Although Cu has been widely accepted as growth promoter due to its antimicrobial effect (Borkow and Gabbay, 2005; Borkow and Gabbay, 2009), consistent data to demonstrate how Cu modulates gut microbiota is still lacking in poultry. Supplementation of high levels (187.5 ppm) of Cu from either CuSO_4_ or Cu hydroxychloride did not alter the number of ileal *Lactobacillus* in broiler chickens (Pang et al., 2009). Increasing the dose of dietary Cu hydroxychloride and CuSO_4_ non-selectively and linearly reduced the population of both beneficial bacteria *Lactobacillus* and pathogenic groups *Bacteroides* and *Enterobacteriaceae* in cecum of broilers (Nguyen et al., 2022), which is similar to a newly weaned pig study in which CuSO_4_ supplementation decreased the counts of *Lactobacilli* and *Enterobacteriaceae* in the cecum (Mei et al., 2010). Inclusion of 36.75 ppm of Cu-bearing montmorillonite in broiler diets reduced the total viable counts of *E. coli* and *Clostridium* in the small intestine and cecum, but CuSO_4_ had no effect (Xia et al., 2004). These findings suggest that different levels and sources of Cu have inconsistent effects on bacteria populations.

Unlike those findings, in the current experiments, 30 ppm Cu-MHAC increased (*p* < 0.05) the relative abundance of beneficial bacteria, *Lactobacillus spp*. *Clostridium Cluster XIVa* and/or *Lactobacillus acidophilus* in comparison to CuSO_4_ and TBCC or negative control, and reduced the abundance of *E. Coli* and/or *Enterobacteriaceae* in comparison to CuSO_4_ or TBCC, 15 ppm Cu-MHAC reduced the percentage of *E. Coli* of total bacteria compared to TBCC.

*Lactobacilli* are present throughout the gastrointestinal tract of poultry and have various biochemical properties, such as producing antibacterial compounds (Gomes and Malcata, 1999) and possessing potential anti-inflammatory and antioxidant activity (Wu et al., 2013; Oh et al., 2018; Li et al., 2019; Talib et al., 2019; Müller et al., 2021). *Clostridium cluster XIVa* includes many known butyrate-producing bacteria and plays beneficial roles in the regulation of intestinal inflammation in experimental mouse models (Van den Abbeele et al., 2013; Onrust et al., 2015). Increase of *Lactobacillus* and *Clostridium cluster XIVa* could potentially improve broiler health by exhibiting antimicrobial, anti-inflammatory and antioxidant benefits. These results revealed the benefits of Cu-MHAC over inorganic forms of Cu, CuSO_4_ or TBCC, by shifting the gut microbiota to more beneficial microflora. This is probably due to the greater bioavailability of Cu-MHAC (Wang et al., 2007) and additional benefits of the chelate ligand, HMTBa, an organic acid form of methionine that exhibits antimicrobial effect (Guo et al., 2022). The improvement of gut morphometry could be the indirect outcome of anti-microbial effect of Cu-MHAC.

## Conclusion

In summary, dietary inclusion of low levels (15–30 ppm) of Cu-MHAC had comparable growth performance as high level of TBCC and CuSO_4_ while improving gut microflora and gut morphometry in broilers subject to coccidiosis vaccination or coccidia challenge. 15–30 ppm Cu-MHAC could be used to replace high levels of CuSO_4_ and or TBCC supplementation in broiler diets and used as a complimentary strategy to improve animal gut health.

## Data Availability

The original contributions presented in the study are included in the article/supplementary material, further inquiries can be directed to the corresponding authors.
